# Integrated transcriptomics–metabolomics analysis reveals biomarkers and metabolic dysregulation characteristics of parenteral nutrition–associated liver disease

**DOI:** 10.3389/fnut.2026.1760274

**Published:** 2026-03-31

**Authors:** Yong Huang, Xiuzhi Yang, Dandan Liu, Songhan Qin, Yifan Cao, Ming Xie, Jiwei Wang

**Affiliations:** 1 Department of General Surgery, Digestive Disease Hospital, Affiliated Hospital of Zunyi Medical University, Zunyi, Guizhou, China; 2 Guizhou Provincial Key Laboratory of Digestive Disease, Zunyi, Guizhou, China

**Keywords:** biomarkers, metabolomics, non-targeted metabolomics, parenteral nutrition-associated liver disease, RNA sequencing

## Abstract

**Background:**

Parenteral nutrition-associated liver disease (PNALD) is the most severe complication of long-term parenteral nutrition. It has a high incidence rate and can cause serious harm to patient health. Biomarkers and metabolites associated with PNALD are poorly characterized. This study aimed to identify biomarkers and key metabolites associated with PNALD progression.

**Methods:**

A PNALD mouse model was established, and liver tissues were collected for RNA sequencing and non-targeted metabolomics. Differentially expressed genes (DEGs) and differentially expressed metabolites (DEMs) were identified. Candidate biomarkers were identified using machine-learning algorithms (least absolute shrinkage and selection operator and support vector machine-recursive feature elimination). Gene set enrichment analysis (GSEA) and immune cell infiltration analysis were conducted. Finally, the expression of identified biomarkers in clinical samples were validated using reverse transcription quantitative polymerase chain reaction.

**Results:**

Histopathological analysis revealed disordered hepatocyte arrangement and mild inflammatory infiltration in PNALD livers, along with significantly increased liver function markers. Transcriptomic and metabolomic analyses revealed 142 DEGs and 18 DEMs. Using the dual machine-learning screening strategy, *Itgam, Clec4d, Orm2, Lcn2*, and *Cd14* were further screened and identified as biomarkers, whereas 6-n-octylaminouracil, 6β-hydroxy-hydromorphone, and α teresantalic acid were identified as key metabolites. GSEA indicated that these biomarkers were enriched in the cytokine-cytokine receptor interaction and oxidative phosphorylation pathways. Immune infiltration analysis revealed positive correlations between the biomarkers and 19 cell types, particularly myeloid-derived suppressor cells. Clinical validation confirmed consistent expression trends for all five biomarkers with prior analyses.

**Conclusion:**

By leveraging machine learning-aided multi-omics integration, this study identified five biomarkers and three key metabolites that provide novel insights into potential therapeutic targets for PNALD.

## 1 Introduction

Parenteral nutrition-associated liver disease (PNALD) is a common hepatobiliary complication that occurs in patients receiving long-term parenteral nutrition support. It primarily manifests as parenteral nutrition-related cholestasis, hepatocellular steatosis, hepatic fibrosis, and even cirrhosis (1–3). These complications not only increase patient morbidity and impair health, but also limit the long-term application of parenteral nutrition therapy (1). Epidemiological studies indicate that the incidence of PNALD is as high as two-thirds in neonates dependent on parenteral nutrition (4). Similarly, in adults with intestinal failure who receive long-term parenteral nutrition, the incidence is 15%−40% (4). Furthermore, in patients of all ages receiving long-term parenteral nutrition, the incidence of mild-to-moderate hepatic fibrosis and steatosis was reported to be 85% and 50%, respectively (5). Currently, known risk factors for PNALD include short bowel syndrome, intestinal bacterial overgrowth and translocation, hepatobiliary circulation disorders, and insufficient enteral nutrition intake (6, 7). However, the underlying pathological mechanism have not been fully elucidated, which has limited the development of clinical intervention strategies. At the therapeutic level, reducing the intake of soybean oil-based enteral nutrition (SO-ILE), or replacing it with non-SO-ILE can reverse PNALD in some cases, although the long-term safety and efficacy remain unclear (8). Several strategies for preventing or reversing PNALD have attracted interest, including promoting intestinal adaptation, advancing enteral feeding, and preventing central venous catheter infection (9, 10). However, most of these strategies lack sufficient support from well-designed prospective clinical trials. Therefore, clarifying the pathogenesis of PNALD and identifying biomarkers of disease progression remain necessary.

The advent of high-throughput sequencing technology has provided a powerful tool for examining complex disease mechanisms. Specifically, RNA sequencing (RNA-seq) enables the comprehensive identification of gene fusions, splice variants, mutations, and differential gene expression (10, 11). It not only plays an important role in deciphering the molecular mechanisms of cancer and formulating diagnosis and therapeutic strategies, but also lays a foundation for investigating the gene regulatory networks of chronic diseases, such as liver diseases (12, 13). Similarly, as a systematic biochemical analysis technique, non-targeted metabolomics can reveal global metabolic changes in biological samples (14). This approach directly reflects the net outcome of interactions across multiple levels, including genetic, transcriptomic, proteomic, and environmental factors. Studies have confirmed that metabolic abnormalities are closely associated with PNALD. For instance, omega-3 polyunsaturated fatty acids, such as eicosapentaenoic acid and docosahexaenoic acid, showed a prophylactic effect against the occurrence of PNALD (15). Conversely, disruptions in lipogenesis, triglyceride secretion, and fatty acid oxidation have been found to drive the pathological progression of PNALD (16). These results suggest that deciphering the metabolomic profiles of PNALD is essential for achieving targeted metabolic intervention. However, current PNALD studies are focused on the gut microbiota or proteome levels, leaving gaps in understanding its pathophysiology and etiology (2). In particular, few studies have integrated transcriptomics and metabolomics to examine PNALD.

To identify reliable diagnostic biomarkers for PNALD, a murine model of PNALD was established in this study. RNA-seq was combined with untargeted metabolomics to screen for differentially expressed genes (DEGs) and differentially expressed metabolites (DEMs). Candidate biomarkers were identified through integrated interaction network analysis and machine-learning approaches. Additionally, bioinformatics analyses were used to elucidate their putative molecular mechanisms involved in PNALD progression, thereby providing evidence to improve the clinical diagnosis and therapy of PNALD.

## 2 Methods

### 2.1 Animal acquisition and model establishment

Thirty male specific pathogen-free (SPF) C57BL/6J mice (8–10 weeks old, 25g−30 g) were purchased from GemPharmatech Co., Ltd. (Jiangsu, China; License No. SCXK (Su) 2023-0009). Mice were housed in an SPF-grade environment, with controlled temperature (22 ± 2 °C), a relative humidity (50% ± 10%), and a 12-h light-dark cycle. During the 7-day acclimatization period, the mice had free access to standard chow and water, and their condition was monitored daily. Following acclimatization, the mice were randomly divided into two groups based on their body weight.

Mice were anesthetized with isoflurane inhalation (4%−5% for induction, 1%−3% for maintenance) with 100% oxygen at a flow rate of 1 L/min, and anesthesia depth was monitored by reflex testing. After induction of anesthesia, a neck incision was made to expose the right internal jugular vein. A venous incision was made, and a catheter pre-filled with heparin solution was inserted. The catheter was then advanced subcutaneously through the neck to the tail and fixed. The incision was sutured, and the catheter was connected to a micropump. After a 2-day postoperative recovery period, the mice underwent a 7-day intervention. The PNALD group received parenteral nutrition through a conscious drug delivery system (KW-CZX, Nanjing Calvin Biotechnology Co., Ltd, Nanjing, China). The parenteral nutrition formulation consisted of fat emulsion, amino acids, and glucose mixed in a 1:2:2 ratio. In contrast, the Control group received a saline infusion along with a standard chow diet. Following drug administration, the mice were anesthetized with isoflurane, and venous blood was collected. The mice were euthanized by cervical dislocation, and the left hepatic lobe was isolated. The wet weight of the liver was measured and preserved for subsequent study. No mice died during the entire experiment. The study was approved by the Animal Ethics Committee of Zunyi Medical University (No. 2024-1-191).

### 2.2 Histological observations

Liver tissues were fixed in 4% paraformaldehyde for 24 h at room temperature. After fixation, the tissues were washed in phosphate-buffered saline and dehydrated through a graded ethanol series (70%, 80%, 95%, and 100%, 1 h each). The samples were then cleared in xylene (two changes, 1 h each) and infiltrated with molten paraffin wax at 56 °C−58 °C (three changes, 1 h each). Finally, the tissues were embedded in paraffin blocks using a standard embedding mold. After embedding, paraffin blocks were sectioned into 4μm−5 μm thick slices using a microtome. Sections were mounted onto glass slides and dried at 60 °C for 1 h−2 h. After deparaffinization and rehydration, the sections were stained with Harris hematoxylin (Sigma-Aldrich, St. Louis, MO, USA) for 5 min, rinsed in running tap water, and differentiated in 1% acid alcohol for 30 s. After rinsing in Scott's tap water for 1 min, the sections were counterstained with 0.5% eosin Y solution (Thermo Fisher Scientific, Waltham, MA, USA) for 3 min. Finally, the sections were dehydrated through a graded ethanol series, cleared in xylene, and mounted with neutral balsam (Sinopharm Chemical Reagent Co., Ltd., Shanghai, China). The tissue morphology was observed under an Olympus CX43 biological microscope (Olympus Corporation, Japan).

### 2.3 Serum biochemical marker detection

Approximately 500 μL−800 μL of venous blood was collected from mice immediately after euthanasia as terminal blood collection. All efforts were made to minimize suffering. Large-volume blood samples were collected only after the animals were confirmed dead under deep anesthesia, in accordance with institutional guidelines for terminal blood collection, which permits the collection of unrestricted blood volumes. Serum was obtained by incubating the samples at 4 °C overnight and centrifuging at 3,500 rpm for 10 min. The aspartate transaminase (AST) kit (Cat no. 701138), alanine aminotransferase (ALT) kit (Cat no. 70111), alkaline phosphatase (ALP) kit (Cat no. 701136), Gamma-glutamyl transferase (GGT) kit (Cat no. 702137), total bile acid (TBA) kit (Cat no. 70917), direct bilirubin (DBIL) kit (Cat no. 70184), and total bilirubin (TBIL) kit (Cat no. 70114) were purchased from Biobase Biodustry (Shandong) Co., Ltd. (Shandong, China) and used for serum biochemical indicator analysis. AST, ALT, ALP, GGT, TBA, DBIL, and TBIL levels were measured using an automatic biochemical analyzer [Biobase Biodustry (Shandong) Co., Ltd, Shandong, China]. Calibration was done using a composite calibration standard before operation, and system stability was validated using a composite quality control (QC) material. Detection parameters were set according to the manufacturer's instructions. Specifically, AST and ALT levels were measured using the continuous monitoring method, ALP and GGT activities were measured using the enzymatic reaction rate method, DBIL and TBIL were measured using the vanadate oxidation method, and TBA concentration was measured using the cyclic enzyme method. After completing the reaction and detection, the absorbance values for each indicator were read, and their concentrations were calculated. Each sample was tested in triplicate, and the average value was recorded.

### 2.4 RNA-seq and data preprocessing

The left hepatic lobe was excised for RNA-seq. Total RNA was isolated and purified using TRIzol reagent (Invitrogen, Carlsbad, CA, USA). RNA concentration and purity of each sample were assessed using a NanoDrop ND-1000 spectrophotometer (NanoDrop, Wilmington, DE, USA). RNA integrity was verified via agarose electrophoresis and a Bioanalyzer 2100 (Agilent, CA, USA). Poly (A) RNA was purified from 1 μg of total RNAand fragmented at 94 °C. Fragmented RNA was reverse-transcribed into cDNA, and cDNA duplexes were generated. After magnetic bead purification, double-stranded cDNA was processed for library construction by polymerase chain reaction (PCR), adapter ligation, and poly (A) tailing, with two-strand synthesis performed. Fragment were sized and purified using magnetic beads. Double-stranded DNA was digested, and PCR was used to generate libraries with an average size of 300 ± 50 bp. Libraries were sequenced on an Illumina NovaSeq™ 6000 (LC-Bio Technology CO., Ltd., Hangzhou, China) with paired-end 150-bp reads. Transcriptomes for all samples were merged to reconstruct a comprehensive transcriptome using gffcompare (https://github.com/gpertea/gffcompare/). Subsequently, mRNA expression levels were estimated using StringTie, with quantification as fragments per kilobase of transcript per million mapped reads (FPKM). The scatterplot3d package (v 0.3–42) was used for principal component analysis (PCA) to standardize the raw data (17). The DEGs between the two groups were obtained by DESeq2 (v 1.38.0; https://bioconductor.org/packages/DESeq2/) with the following criteria: log2 Fold Change (FC)| > 4, and *p* < 0.01 (18). To explore the functions of the DEGs, Gene ontology (GO), Kyoto encyclopedia of genes and genomes (KEGG) enrichment, and gene set enrichment analysis (GSEA) were performed using the clusterProfiler package (v 4.2.2) (19).

### 2.5 Identification of candidate genes

To investigate the interactions of DEGs at the protein level, a protein-protein interaction (PPI) network was constructed using the STRING database (https://string-db.org/) with an interaction score >0.4. The resulting network was visualized using Cytoscape (v 3.9.1) (20). The top 30 genes from each of the three algorithms (degree, closeness, and betweenness) in the cytoHubba plugin of Cytoscape (v 3.9.1) were identified. Candidate genes were then identified by taking the intersection of the top 30 genes using the plotrix package (v 3.8–4).

### 2.6 Identification of candidate biomarkers

To obtain candidate biomarkers, the least absolute shrinkage and selection operator (LASSO) and support vector machine-recursive feature elimination (SVM-RFE) algorithms were applied to the sequencing data. First, the LASSO regression analysis was performed on the candidate genes using the glmnet package (v 4.1.4) (21). To enhance the interpretability of the model and the reproducibility of the results, the elastic net mixing parameter was set to α = 1 (L1 regularization) with a fixed random seed (seed = 604). The optimal regularization parameter lambda.min was determined through 5-fold cross-validation (cv. glmnet function), and the genes with non-zero coefficients corresponding to lambda.min were selected as feature genes. The SVM-RFE analysis was performed using the e1071 package (v4.7-1.1) (22). The kernel function of the SVM model was set to linear, and feature importance was iteratively evaluated via 5-fold cross-validation while gradually removing the least important features in each iteration. The optimal feature subset was defined as the set that yielded the minimum cross-validation error rate during this iterative process. Then, candidate biomarkers were acquired by overlapping the gene sets in two algorithms using the ggvenn package (v 1.7.3) (23). The correlation between candidate biomarkers was evaluated by Spearman's analysis via the psych package (v 2.2.9) (24).

### 2.7 Immune infiltration analysis

The infiltration abundance of 28 immune cells between the PNALD and control samples in the RNA-seq data was estimated using the ssGSEA algorithm (25). Due to the fact that the standard gene sets for the ssGSEA algorithm are constructed based on human genes, this study first utilized the R package biomaRt (v 2.56.1) (26) to homologously map mouse gene symbols to their corresponding human genes. Subsequently, the FPKM expression matrix was converted into a suitable input matrix for analysis, and the gsva () function (with method = “ssgsea” specified) was used to calculate the enrichment scores of immune cells, which were then standardized. By comparing the standardized enrichment scores between groups, differential immune cells (DICs) were screened, and their significance was analyzed using the Wilcoxon rank-sum test. The analysis results were visualized using the ggplot2 package (v 3.4.1). The correlation between biomarkers and DICs, as well as between DICs, was calculated by Spearman correlation analysis via the psych package (v 2.2.9). Finally, the GeneMANIA database (http://genemania.org/) was used to identify genes related to the biomarker functions.

### 2.8 Construction of molecular regulatory network

The transcription factors (TFs) linked to biomarkers were predicted using the TRRUST database (https://www.grnpedia.org/trrust/) of the NetworkAnalyst platform (https://www.networkanalyst.ca/). Subsequently, a two-step prediction was performed using the multiMiR package (v 1.12.0; https://bioconductor.org/packages/multiMiR/) (27): First, biomarkers-associated microRNAs (miRNAs) were predicted in the miRDB database (http://www.mirdb.org/), yielding a unique list of miRNAs. Then, long non-coding RNAs (lncRNAs) with regulatory relationships to the aforementioned miRNAs were predicted in the miRTarBase database (https://mirtarbase.cuhk.edu.cn/~miRTarBase/). The results were visualized by Cytoscape (v 3.9.1).

### 2.9 Prediction of drugs

To predict potential therapeutic drugs associated with the biomarkers, this study utilized the Drug-Gene Interaction Database (DGIdb, https://www.dgidb.org/) for analysis. The list of target genes was directly input into the database for searching, employing its default parameters and criteria. The resulting drug-gene interaction network was constructed using Cytoscape software (v 3.9.1).

### 2.10 Non-targeted metabolomics and data preprocessing

After grinding, the liver samples were incubated at −20 °C for 30 min to precipitate the proteins. Following centrifugation at 20,000 × g for 10 min at 4 °C, the supernatant was collected and centrifuged for 5 min. The resulting supernatant was transferred to a new vial for ultra-performance liquid chromatography-high-resolution mass spectrometry analysis. QC samples were prepared by mixing equal volumes of the supernatants from all of the samples. An ACQUITY UPLC HSS T3 column (100 mm × 2.1 mm, 1.8 μm; Waters) was used for separation. The mobile phase consisted of phase A (5 mmol/L ammonium acetate + 5 mmol/L acetic acid + water) and phase B (acetonitrile). Gradient elution was performed as follows: 0~0.8 min, 2% B; 0.8~2.8 min, 2%~70% B; 2.8~5.6 min, 70%~90% B; 5.6~6.4 min, 90%~100% B; 6.4~8.0 min, 100% B; 8.0~8.1 min, 100%~2% B; 8.1~10.0 min, 2% B. The flow rate was 0.35 ml/min. The injection volume for each sample was 4 μL. The column oven was maintained at 40 °C. For mass spectrometry, a high-resolution tandem mass spectrometer (Triple TOF 6600, AB SCIEX, USA) was used to detect metabolites eluted from the column. Each sample was analyzed in both positive and negative electrospray ionization modes. The ESI temperature was 500 °C. The voltage was + 5,000 volts in positive ion mode and −4,500 volts in negative ion mode. The curtain gas pressure of the ion source was 30 psi. Gas 1 (auxiliary gas) and Gas 2 (sheath gas) pressures are both set to 60 psi. Mass spectrometric data were obtained using full scan and information-dependent acquisition (IDA) modes. In one acquisition cycle, the full scan acquisition range was 60–1,200 Da, with a scan time of 150 ms. Based on the full scan, the top 12 signal ions with an accumulation intensity greater than 100 were selected for IDA scanning. For the IDA scans, the acquisition range was set to 25–1,200 Da, with an acquisition time of 30 ms per scan. Dynamic exclusion was set to 4 s. The LC–MS raw data files were converted into mzXML format and processed by the XCMS, CAMERA and metaX toolbox programs of R software (v 4.0). Each ion was identified by combining retention time and m/z data. The intensities of each peak were recorded and a three-dimensional matrix containing arbitrarily assigned peak indices, sample names and ion intensity information. Molecular mass data for the samples were matched against database entries, with metabolite annotation performed using the KEGG (https://www.kegg.jp) and HMDB (https://www.hmdb.org/) databases. Validation of metabolite identification was performed using an internal metabolite fragment spectrum library. The metabolite data excluded samples with missing values exceeding 80% or QC samples with missing data exceeding 50%. Data interpolation was conducted using the K-nearest neighbors' method, and data normalization was achieved through probability quotient normalization.

### 2.11 Identification and enrichment analysis of DEMs

PCA was performed using the scatterplot3d package (v 0.3-42) to visualize and standardize the non-targeted metabolomics data. Orthogonal partial least squares-discriminant analysis (OPLS-DA) was conducted using the ropls package (v 1.30.0) (28). The variable importance in projection (VIP) values of the metabolites from the OPLS-DA analysis were acquired. Finally, the permutation test method was used to verify whether the OPLS-DA model had overfitting. The specific procedure was as follows: the sample group labels were randomly permuted 200 times, and for each permutation, the OPLS-DA model was reconstructed and its predictive ability parameter (Q^2^Y) was calculated. The criteria for a valid model were as follows: the Q^2^Y value of the original model was significantly higher than that of the permutated distribution (*p* < 0.05), and the intercept of the Q^2^Y regression line with the Y-axis was less than 0.05. Based on this, the ggstatsprot package was utilized to perform *t*-test in all samples of non-targeted metabolomics data. Metabolites that meet the threshold of VIP >2.5 and *p* < 0.05 were considered DEMs. The MetaboAnalyst database (http://www.metaboanalyst.ca/) was used to examine DEM-related pathways.

### 2.12 Identification of candidate metabolites

To obtain candidate metabolites, the LASSO and SVM-RFE algorithms were applied to the non-targeted metabolomics data. First, the LASSO algorithm with 5-fold cross-validation was applied using the glmnet package (v 4.1.4), and SVM-RFE algorithms with 5-fold cross-validation were applied using the e1071 package (v 1.7-13). Candidate metabolites were identified by the overlap of metabolites in two algorithms using the ggvenn package (v 1.7.3). The correlation between candidate metabolites was explored by Spearman's analysis using the psych (v 2.2.9) with the |cor| >0.3 and *p* < 0.05.

### 2.13 Correlation analysis between candidate metabolites and candidate biomarkers

Spearman correlation coefficients between candidate metabolites and biomarkers in all samples of the RNA-seq data and metabolome sequencing data were obtained through the psych package (v 1.7.3) (|cor| > 0.3, *p* < 0.05). When a metabolite was strongly correlated with a biomarker, the metabolites and genes were considered key metabolites and biomarkers. Cytoscape (v 3.9.1) was utilized to draw a network of key metabolites and biomarkers.

### 2.14 Expression of biomarkers and key metabolites

The expression of biomarkers in the RNA-seq data and the expression of key metabolites between the PNALD and control samples in the non-targeted metabolomics data were compared by the Wilcoxon test (*p* < 0.05), and the results were displayed using the ggplot2 package (v 3.4.1).

### 2.15 Reverse transcription quantitative PCR

Reverse transcription quantitative PCR (RT-qPCR) was used to measure the expression of the candidate biomarkers in tissue samples. Total RNA was extracted using TRIzol reagent (Vazyme, Nanjing, China). RNA concentrations were determined using a NanoPhotometer N50 (Implen, Munich, Germany). The mRNA was reverse transcribed into cDNA using a test kit (Yi Sheng, Wuhan, China). Finally, RT-qPCR was conducted on the candidate biomarkers. The expression of the biomarkers between the PNALD and control samples was calculated by the 2^−ΔΔ*Ct*^ method, and the gene expression differences were sized using a Student's *t*-test. The detailed information on the primers and machine testing conditions was listed in Supplementary Table 1.

### 2.16 Statistical analysis

Bioinformatics analyses were conducted using the R programming language (v 4.2.2). The Wilcoxon test or Student's *t*-test was used to compare the disparities between two groups. Graphpad Prism software (v 10.1.2) was used to achieve statistical analysis and visualization (29). For comparisons between two groups, an unpaired two-tailed Student's *t*-test was used if the data were normally distributed with equal variances; otherwise, the Mann-Whitney *U* test was applied. The data are expressed as the mean ± standard error of the mean (SEM). A *p*-value < 0.05 was considered statistically significant.

## 3 Results

### 3.1 Effects of PNALD on mouse liver function

**Figure 1:**
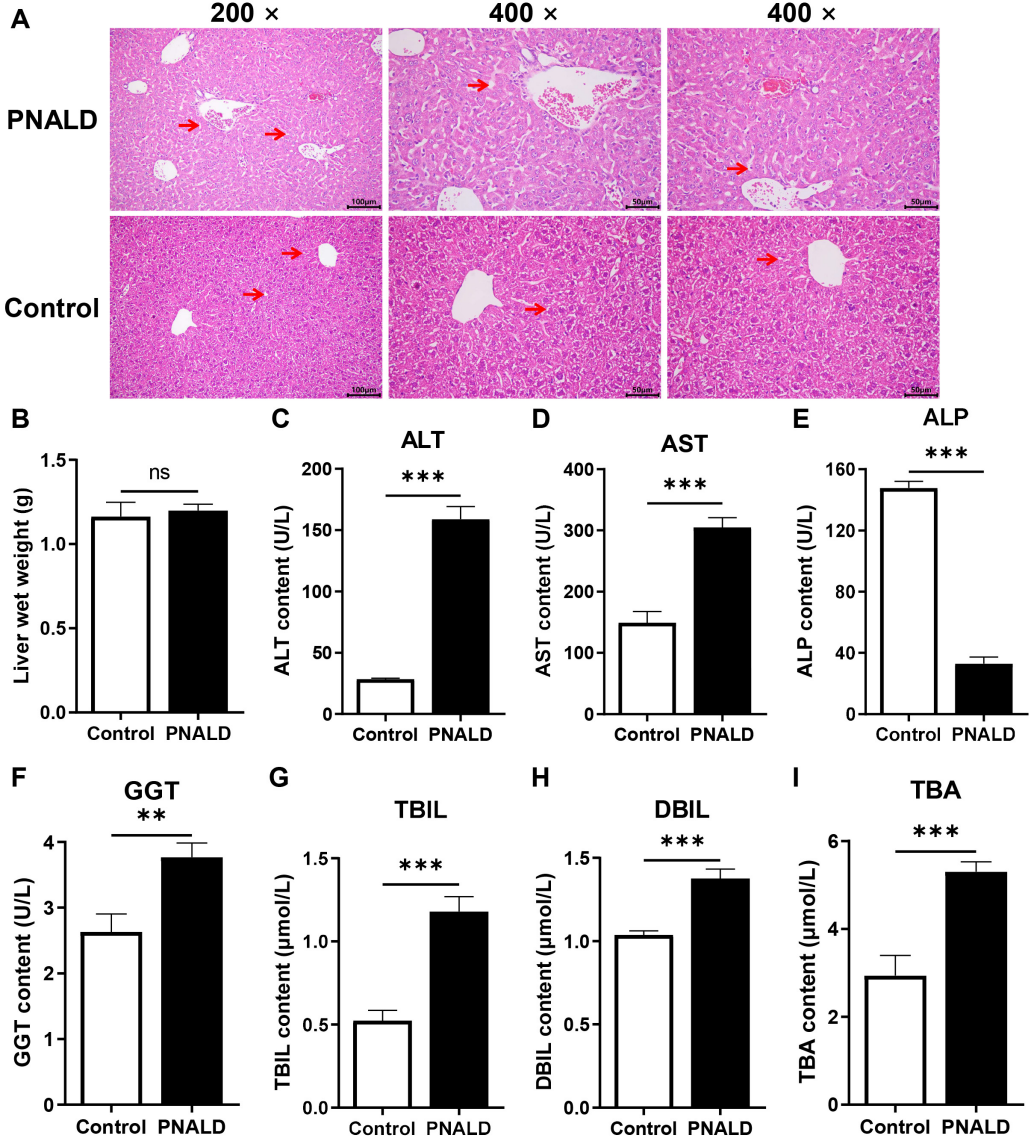
Hepatic pathology and liver function marker analysis in a PNALD mouse model. **(A)** Representative H&E-stained liver tissue sections. The red arrows indicate fatty vacuolar degeneration. Scale bar indicates 50μm. **(B)** Liver wet weight. **(C–I)** Automatic biochemical analysis of liver function indicators, including Alanine aminotransferase (ALT) **(C)** Aspartate transaminase (AST) **(D)** Alkaline phosphatase (ALP) **(E)**. Gamma-glutamyl transferase (GGT) **(F)** Total bilirubin (TBIL) **(G)** Direct bilirubin (DBIL) **(H)** Total bile acids (TBA) **(I)** ***indicates *p* < 0.001; **indicates *p* < 0.01. NS, not significant. PNALD, parenteral nutrition–associated liver disease; H&E, hematoxylin-eosin.

To determine the effect of PNALD on the liver, morphological analyses and liver function tests were performed in the control and PNALD groups. The results showed that the histology of the livers of the PNALD group was different from that of the control mice. Hematoxylin–eosin (H&E) staining of the livers in the control group revealed that the liver lobule structure was intact, the hepatocytes were arranged regularly in a radial pattern centered on the central vein, with no obvious pathological changes evident (Figure 1A). The PNALD group exhibited disorganized hepatocytes with a small number of infiltrating inflammatory cells. There was no significant difference in liver wet weight; however, the liver function indicators were significantly abnormal (Figure 1B). ALT and AST levels, which reflect hepatocyte injury (Figures 1C, D), ALP and GGT levels, which reflect cholestasis (Figures 1E, F), TBIL and DBIL, which reflect bilirubin metabolism (Figures 1G, H), and TBA, which reflects bile acid metabolism (Figure 1I), were all significantly increased. Taken together, PNALD significantly induces histological damage in the liver, and causes hepatocyte injury, cholestasis, and abnormalities in bilirubin and bile acid metabolism, which were manifested as elevated liver function indicators.

### 3.2 RNA-seq identifies potential candidate biomarkers for PNALD

**Figure 2:**
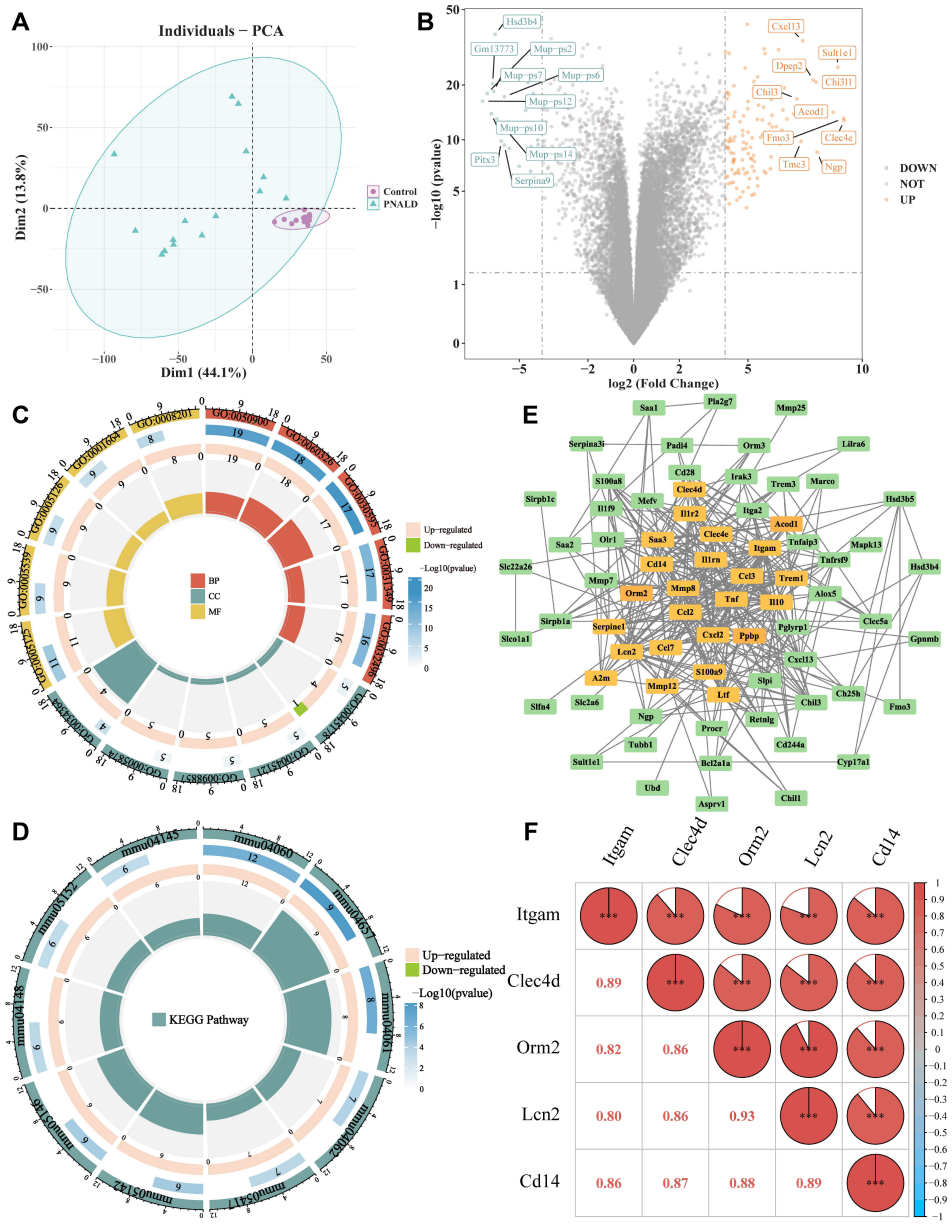
Differential gene expression and biomarker identification in a PNALD mouse model. **(A)** Principal component analysis (PCA) plot showing differences between PNALD and control samples. **(B)** Volcano plot of differentially expressed genes (DEGs). Orange dots represent upregulated DEGs, blue dots represent downregulated DEGs, and gray dots represent non-differentially expressed genes. **(C)** Circular graph of gene ontology (GO) enrichment analysis, displaying enriched biological processes (BP), cellular components (CC), and molecular functions (MF). **(D)** Kyoto encyclopedia of genes and genomes (KEGG) pathway enrichment analysis of the DEGs. **(E)** Protein-protein interaction network of DEGs, with nodes representing genes and edges representing interactions. **(F)** Correlation heatmap showing the correlations among the candidate biomarkers. The color intensity and values indicate the correlation strength. ***indicates *p* < 0.001. PNALD, parenteral nutrition–associated liver disease.

To elucidate the molecular regulatory mechanisms underlying the progression of PNALD and to identify potential biomarkers, RNA-seq was performed on liver samples from the PNALD and control mice. PCA results indicated a clear differentiation between the PNALD and control samples (Figure 2A). A total of 142 DEGs were identified, of which 120 were upregulated in the PNALD groups (Figure 2B). GO analysis identified 679 biological processes including cell chemotaxis (GO:0060326), 23 cellular components such as anchored component of membrane (GO:0031225), and 73 molecular functions including cytokine receptor binding (GO:0005126), which were enriched. This suggests that DEGs participate in PNALD pathogenesis by regulating processes such as cell migration, membrane structure function, and cytokine signaling (Figure 2C, Supplementary Table 2). A KEGG analysis revealed that the DEGs were enriched in 42 pathways, including cytokine-cytokine receptor interaction (mmu04060) (Figure 2D, Supplementary Table 3). The results indicate that abnormal activation of cytokine pathways may represent a key molecular mechanism underlying PNALD-induced liver injury.

Based on the DEGs and functional enrichment analyses, core regulatory genes were further identified through PPI network analysis. Interactions were evident among the DEGs, such as *Cel2, Tnf* , and *Ccl3* (Figure 2E), and 24 candidate genes were obtained (Supplementary Figure 1A). To enhance the specificity and reliability of the candidate biomarkers, the SVM-RFE algorithm was applied, which ultimately identified nine genes (*Itgam, Trem1, Clec4d, Orm2, Lcn2, Cd14, Clec4e, Mmp8*, and *S100a9*) (Supplementary Figure 1B). Subsequently, the LASSO algorithm (optimal lambda value = 0.0001056373) revealed seven genes (*Itgam, Lcn2, Serpine1, Cd14*, Saa3, *Clec4d*, and *Orm2*) (Supplementary Figure 1C). Then, five candidate biomarkers (*Itgam, Clec4d, Orm2, Lcn2*, and *Cd14*) were acquired from the intersection of the gene sets and the two algorithms (Supplementary Figure 1D). A correlation analysis revealed positive correlations among the candidate biomarkers. For example, *Clec4d* (cor = 0.89, *p* < 0.001) was significantly positively correlated with *Itgam* (Figure 2F). These biomarkers may participate in the pathological process of PNALD through shared regulatory networks.

### 3.3 Metabolomics analysis reveals metabolic dysregulation characteristics and candidate metabolites in PNALD

**Figure 3:**
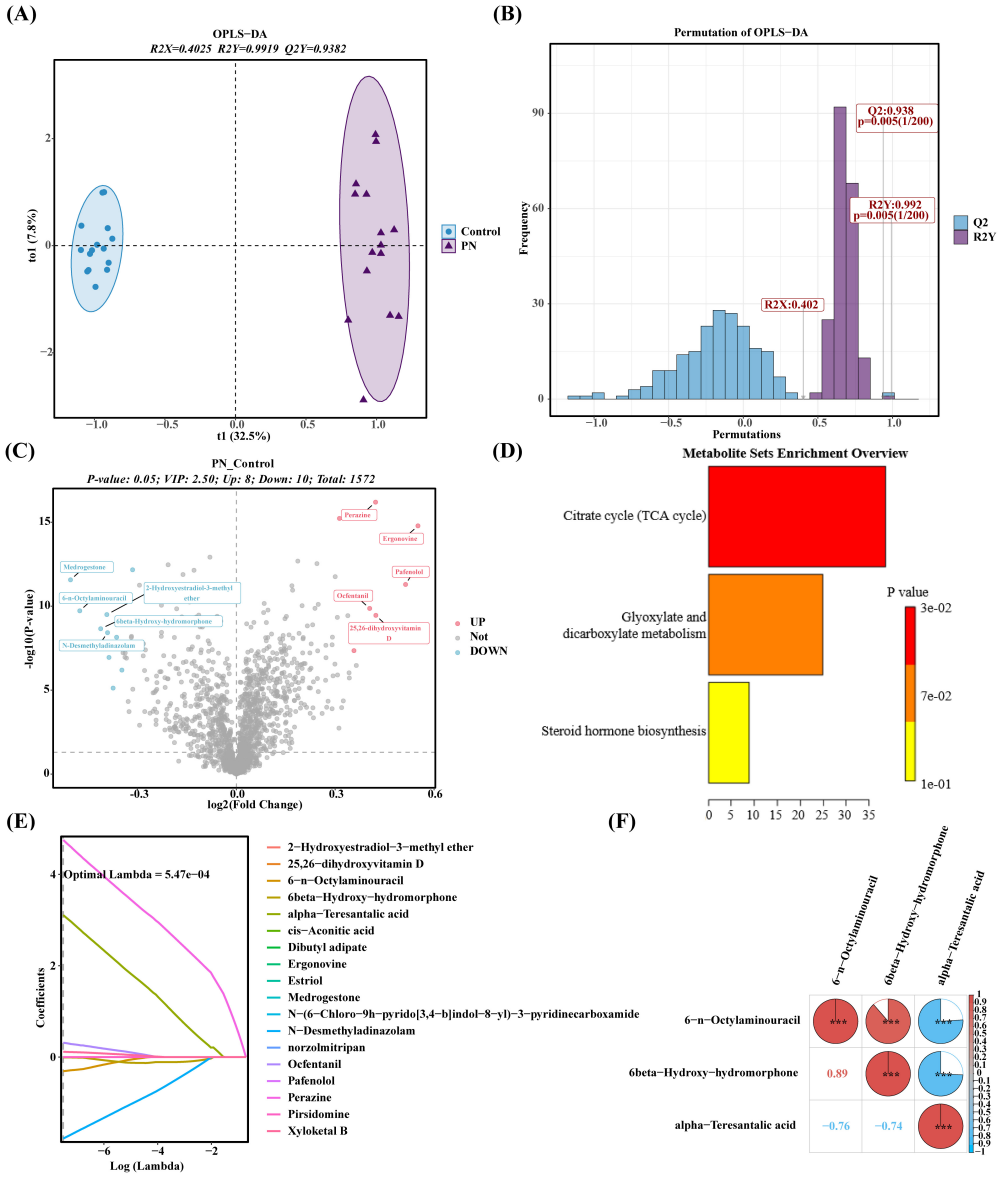
Metabolomic profiling and candidate metabolite identification in a PNALD mouse model. **(A)** Orthogonal partial least squares-discriminant analysis (OPLS-DA) score plot showing separation between PNALD and control samples. **(B)** Permutation test of OPLS-DA, with the x-axis representing permutations and the y-axis representing frequency. **(C)** Volcano plot of differential metabolites (DEMs). Red dots represent upregulated DEMs, blue dots represent downregulated DEMs, and gray dots represent non-differential metabolites. **(D)** Metabolic pathway enrichment analysis of DEMs, with color intensity indicating p value. **(E)** Coefficient profile plot from the least absolute shrinkage and selection operator (LASSO) analysis, with the x-axis being log(lambda) and the y-axis showing coefficients. **(F)** Correlation heatmap showing the correlations among candidate metabolites. Color intensity and values indicate correlation strength and direction (positive or negative). ***indicates *p* < 0.001. PNALD, parenteral nutrition–associated liver disease.

To analyze the metabolic dysregulation characteristics during the occurrence and development of PNALD, non-targeted metabolomics was applied to the mouse liver samples. The OPLS-DA results indicated that the PNALD and control samples were distinguished, with clear separation on the score plot (Figure 3A). The R2Y and Q2Y values were high, indicating that the model had good predictive ability (Figure 3B). A total of 18 DEMs were identified, of which 8 were upregulated in the PNALD groups (VIP > 2.5, *p* < 0.05) (Figure 3C). Three pathways including the citrate cycle (TCA cycle), glyoxylate, steroid hormone biosynthesis and dicarboxylate metabolism, were enriched by DEMs (Figure 3D). The key metabolites with specificity and reliability were further identified through a multi-algorithm screen. The SVM-RFE algorithm identified three metabolites, including 6-octylaminouracil, 6β-hydroxy-morphanone, and α-naphthoic acid (Supplementary Figure 2A), and seven metabolites (6-n-octylaminouracil, 6β-hydroxy-hydromorphone, α-teresantalic acid, N-desmethyladinazolam, ocfentanil, perazine, and xyloketal B) were selected by the LASSO algorithm (Figure 3E). Three candidate metabolites, including 6-n-octylaminouracil, 6β-hydroxy-hydromorphone, and α-teresantalic acid, were acquired from the intersection of metabolites and the two algorithms (Supplementary Figure 2B). Correlation analysis revealed that 6-n-octylaminouracil was positively correlated with 6β-Hydroxy-hydromorphone, and negatively correlated with α-teresantalic acid (Figure 3F). Taken together, we identified three core candidate metabolites, which may participate in the metabolic regulation of PNALD through synergistic or antagonistic interactions.

### 3.4 Correlation analysis of core biomarkers and candidate metabolites in PNALD

**Figure 4:**
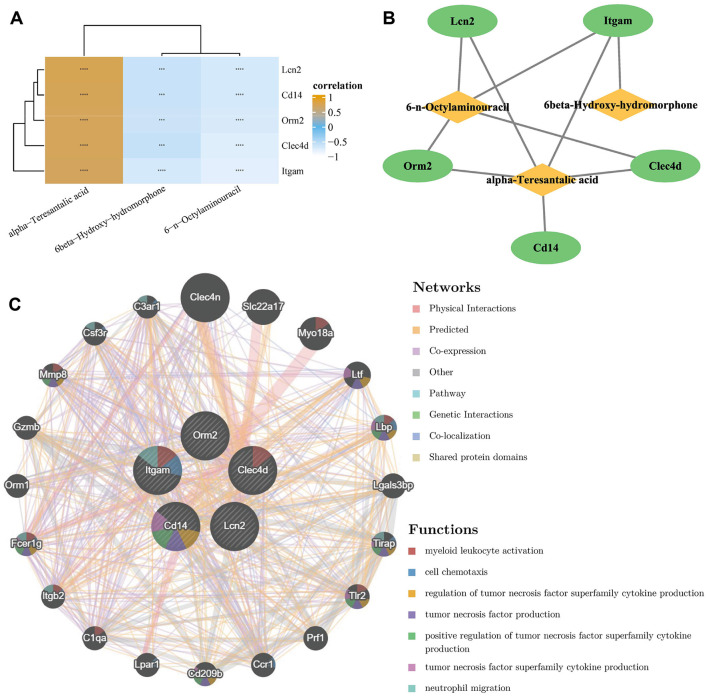
Integrative analysis of candidate metabolites and genes in a PNALD mouse model. **(A)** Heatmap showing the correlations between candidate metabolites and genes. Color intensity indicates correlation strength. **(B)** Network interactions between candidate metabolites and genes. Nodes represent metabolites or biomarkers, and edges represent interactions. **(C)** Network diagram of the top 20 genes with the strongest correlation to biomarkers. Nodes represent genes, and edges represent correlations. Colored segments in the nodes indicate enriched functions, and the legend on the right details the network interaction types and enriched functions. ***indicates *p* < 0.001; ****indicates *p* < 0.0001. PNALD, parenteral nutrition–associated liver disease.

A correlations between candidate metabolites and biomarkers indicated that *Itgam, Clec4d, Orm2, Lcn2*, and *Cd14* showed significant correlations with 6-n-octylaminouracil, 6β-hydroxy-hydromorphone, and α-teresantalic acid (Figure 4A, Supplementary Table 4). An association network was constructed using |Cor| > 0.7 and *p* < 0.05 as screening thresholds. Results revealed that α-teresantalic acid exhibited interactions with five biomarkers, and 6-n-octylaminouracil had interactions with *Itgam, Clec4d, Orm2*, and *Lcn2*. However, 6β-hydroxy-hydromorphone only had interactions with *Itgam* (Figure 4B). The GSEA results indicated that *Itgam, Clec4d, Orm2, Lcn2*, and *Cd14* were all enriched in pathways such as cytokine-cytokine receptor interaction and oxidative phosphorylation (Supplementary Figures 3A–E, Supplementary Table 5). The top 20 genes with the strongest correlation with the biomarkers included *Clec4n, Slc22a17*, and *Myo18a*, and the functions enriched by these genes included cell chemotaxis (Figure 4C). These results indicate that these candidate genes may have an impact on these processes.

### 3.5 Identification of differentially immune cells and correlation analysis with biomarkers in PNALD

**Figure 5:**
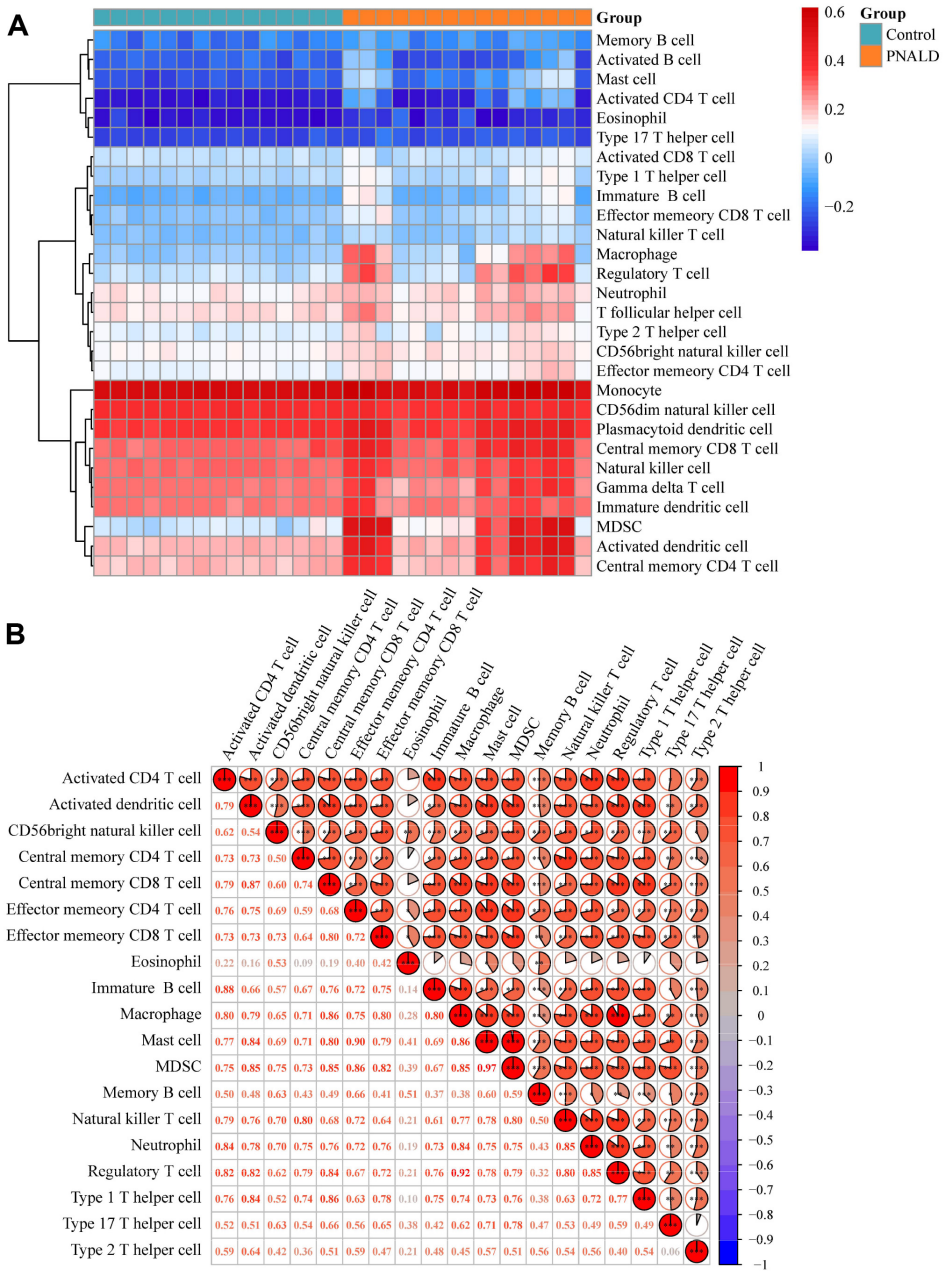
Immune cell infiltration and correlation analysis in a PNALD mouse model. **(A)** Heatmap depicting the abundance of immune cells in the PNALD and control samples. Color intensity represents relative abundance, and the color scale on the right indicates abundance levels. **(B)** Correlation heatmap showing the correlations among the differentially infiltrating immune cells. Color intensity and values indicate correlation strength. *indicates *p* < 0.05; **indicates *p* < 0.01; ***indicates *p* < 0.001. PNALD, parenteral nutrition–associated liver disease.

Immune cell infiltration is a core pathological feature of PNALD progression. Subsequent analyses examined differences in immune cell composition among the samples to identify associations between DICs and core biomarkers. The abundance of 28 immune cell types between the PNALD and control samples revealed that the abundance of monocytes was relatively high in all samples (Figure 5A). Next, 19 DICs such as macrophages and CD56 bright natural killer cells were obtained (Supplementary Figure 4A). The correlation analyses revealed that the correlation between the DICs was positive (Figure 5B). For example, macrophages were positively correlated with regulatory T cells (cor = 0.92, *p* < 0.001). The correlation between biomarkers and these cells indicated that myeloid-derived suppressor cells (MDSCs) had a positive correlation with *cd14* (cor = 0.91, *p* < 0.001), *Clec4d* (cor = 0.90, *p* < 0.001), *Lcn2* (cor = 0.94, *p* < 0.001), *Itgam* (cor = 0.85, *p* < 0.001), and *Orm2* (cor = 0.82, *p* < 0.001) (Supplementary Figures 4B–F, Supplementary Table 6). These results confirmed the high monocyte infiltration characteristic in PNALD samples and identified 19 key DICs, thus indicating a strong positive correlation between the core biomarkers and the MDSCs.

### 3.6 Prediction of upstream molecules and drugs related to biomarkers

Further analysis of the upstream regulatory mechanisms associated with the biomarkers was conducted, and potential therapeutic drugs targeting these biomarkers were identified. The TFs, Jun and Sp1 were linked to *Cd14*, and Foxo3, and Nfkb1 were associated with *Lcn2*. Nfkb1 was associated with *Itgam* (Supplementary Figure 5A). MiRNAs such as mmu-miR-466j, were associated with *Itgam* and *Clec4d*, mmu-miR-7673-3p was associated with *Cd14*, and mmu-miR-6340 was associated with *Lcn2*. LncRNAs such as Taf4 were associated with the miRNAs (Supplementary Figure 5B). Therefore, the role of these factors related to three biomarkers during the progression of PNALD may warrant further exploration. A total of 12 drugs including liarozole were associated with *Itgam*, and 11 drugs, such as IC14, were predicted to be linked to *Cd14*. Docetaxel anhydrous was associated with *Orm2*, but no drugs were predicted to be linked to *Clec4d* and *Lcn2* (Supplementary Figure 5C). The results indicate that these drugs may exhibit activity against PNALD.

### 3.7 Validation of expression levels for biomarkers and key metabolites

**Figure 6:**
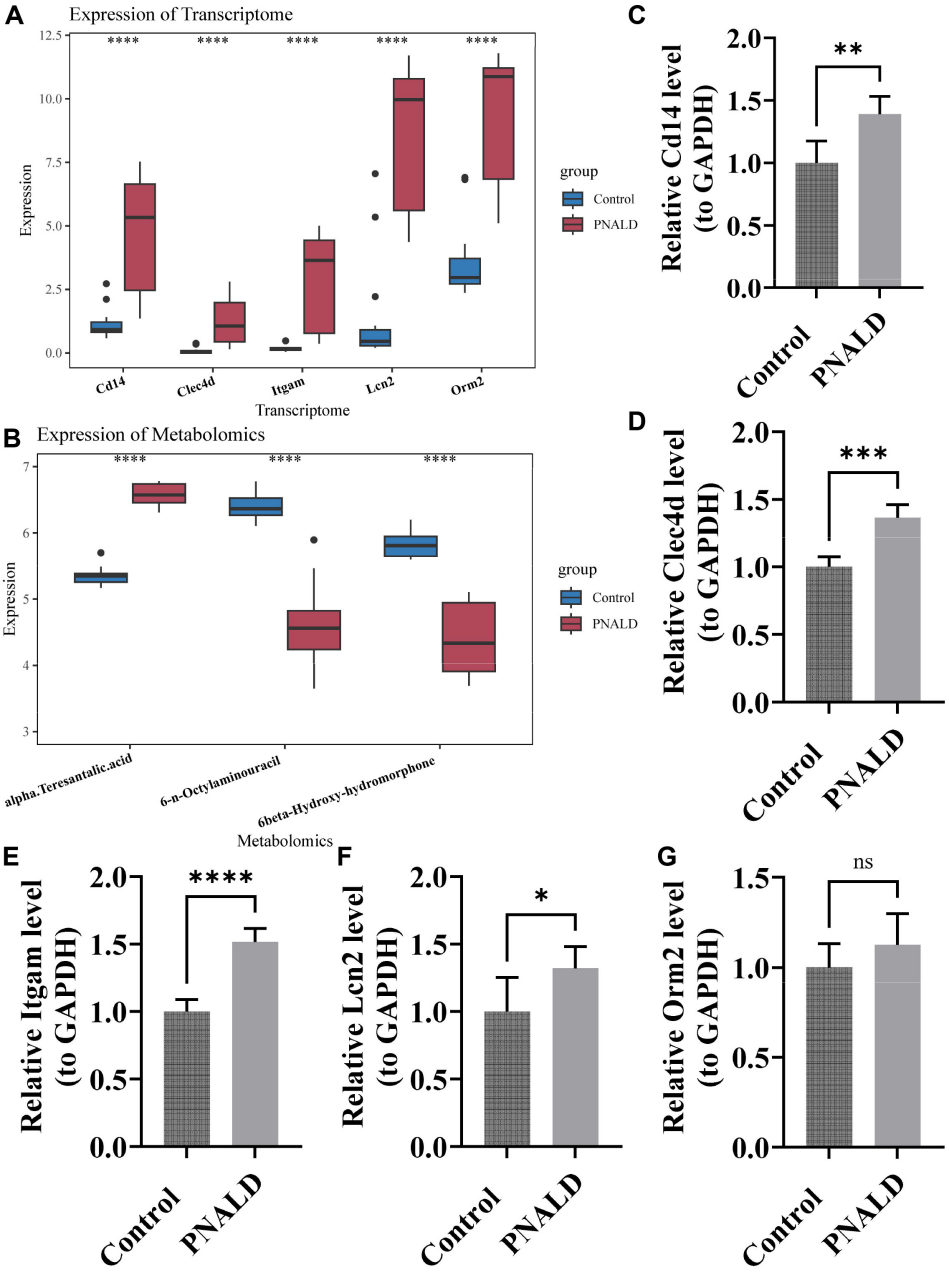
Verification of biomarker and metabolite expression in PNALD. **(A)** Boxplot showing the expression levels of five biomarkers at the transcriptome level in the control and PNALD samples. **(B)** Boxplot showing the expression levels of metabolites in the control and PNALD samples. **(C–G)** Bar graphs showing the relative expression (normalized to GAPDH) of Cd14 **(C)**, Clec4d **(D)**, Itgam **(E)**, Lcn2 **(F)**, and Orm2 **(G)** in control and PNALD samples detected by RT-qPCR. *indicates *p* < 0.05; **indicates *p* < 0.01; ***indicates *p* < 0.001; ****indicates *p* < 0.0001; NS, not significant; PNALD, parenteral nutrition–associated liver disease; GAPDH, glyceraldehyde-3-phosphate dehydrogenase.

The expression of five biomarkers was upregulated in PNALD (Figure 6A). The level of α-teresantalic acid was upregulated, and the expression of 6-n-octylaminouracil and 6β-hydroxy-hydromorphone levels were decreased in PNALD (Figure 6B). The RT-qPCR results confirmed that *Itgam, Clec4d, Lcn2*, and *Cd14* were upregulated in PNALD, validating our previous findings (Figures 6C–G).

## 4 Discussion

PNALD is a common complication in patients receiving long-term parenteral nutrition support, and is primarily characterized by cholestasis, hepatocellular injury, and abnormal liver function (2). Severe cases may progress to liver cirrhosis or liver failure (2). The incidence of PNALD is 40%−60% in pediatric patients and 15%−30% in adult patients receiving long-term parenteral nutrition (2, 30). However, its pathological mechanisms remain incompletely elucidated, and clinical practice lacks specific diagnostic and targeted biomarkers (31). Multi-omics techniques are effective tools for deciphering liver disease mechanisms and screening biomarkers (32). Based on this, the present study systematically explored the molecular regulatory mechanisms of PNALD with transcriptomics, metabolomics, and bioinformatics prediction on mouse models, aiming to provide new insights for disease diagnosis and intervention. Ultimately, this study demonstrated that the pathological mechanisms of PNALD involve the synergistic regulation of inflammatory activation, metabolic disorder, and immune imbalance.

In the present study, the livers of PNALD group mice exhibited obvious pathological damage, characterized by disordered hepatocyte arrangement and accompanied by inflammatory cell infiltration. The levels of ALT and AST, which reflect hepatocyte damage, were significantly elevated, suggesting that PNALD could directly disrupt hepatocyte integrity. The increased levels of ALP and GGT, indicators of cholestasis, indicated impaired biliary transport function. Abnormalities in TBIL and DBIL related to bilirubin metabolism along with increased TBA, which is associated with bile acid metabolism, further confirmed that PNALD could disrupt the core metabolic pathways. Notably, there was no significant difference in liver wet weight between the two groups, implying that early damage during PNALD primarily manifests as functional abnormalities, with morphological changes not yet progressing enough to affect overall liver weight. This is consistent with the clinical finding that PNALD patients often present with asymptomatic liver dysfunction at the early stages (33). Previous studies have identified cholestasis, inflammatory activation, and oxidative stress as core pathological processes in PNALD (34, 35). These findings validated the conserved pathological phenotype of PNALD, establishing a phenotypic anchor to support subsequent molecular mechanism investigations.

To identify the molecular regulatory targets of PNALD, this study employed RNA-seq and identified 142 DEGs. Among these, 120 were upregulated in the PNALD group and enriched in pathways such as cell chemotaxis and cytokine-cytokine receptor interaction. These pathways mediate immune cell migration to damaged areas, representing key steps in the exacerbation of liver inflammation. This is consistent with the fingdings of inflammasome activation observed in murine non-alcoholic fatty liver disease (NAFLD) (36). Through multiple algorithms combining PPI analysis, SVM-RFE and LASSO, followed by RT-qPCR validation, five core biomarkers including *Itgam, Clec4d, Orm2, Lcn2*, and *Cd14*, were finally identified. All these biomarkers are associated with liver inflammation and injury repair. *Itgam* (macrophage integrin αM, also known as CD11b) is a surface marker of monocytes/macrophages and a key molecule for immune cell adhesion and migration. Its overexpression can promote macrophage infiltration and renal fibrosis (37). *Clec4d* belongs to the Dectin-2 subfamily of the C-type lectin receptor family, primarily expressed on myeloid cells and participates in pathogen defense (38). A monocyte subtype with high *Clec4d* expression can activate hepatic stellate cells to exacerbate liver fibrosis (39). *Orm2* is a key regulator of hepatic lipid synthesis. In obese mouse models and patients with NAFLD, the levels of *ORM2* in the liver and plasma are significantly reduced, and its absence disrupts lipid homeostasis (40, 41). In the present study, we found that *Orm2* was upregulated in the liver of PNALD mice. The expression pattern was opposite to the downregulation of *Orm2* observed in classic NAFLD models and patients, suggesting that its upregulation in PNALD represents a compensatory adaptive response of the liver to the specific metabolic disorders of PNALD (42). *Lcn2* (lipocalin 2), a sensitive marker of liver inflammation, exacerbates hepatocyte damage by regulating iron metabolism in cholestatic liver disease (43, 44). *Cd14* is a co-receptor for lipopolysaccharide and can activate the TLR4/NF-κB pathway to trigger the innate immune response of macrophages and monocytes against pathogens and tissue damage (45, 46).

The liver is the metabolic center of the body, and the interference of PNALD with liver metabolism constitutes a crucial part of its pathological mechanism. Using untargeted metabolomics, this study found that samples from the PNALD and control groups could be completely distinguished, with the model demonstrating good predictive ability, confirming significant hepatic metabolic reprogramming in PNALD. A total of 18 DEMs were identified, 8 of which were upregulated. These DEMs were enriched in several pathways including the TCA cycle, steroid hormone biosynthesis, and glyoxylate and dicarboxylate metabolism. The TCA cycle is the core pathway in hepatic energy metabolism. Its disruption in PNALD directly interferes with the energy supply to hepatocytes, exacerbating hepatocyte damage and bile transport dysfunction, thereby forming a vicious cycle of metabolic abnormality and functional impairment (47, 48). Abnormalities in the steroid hormone biosynthesis pathway reflect impaired hepatic detoxification and endocrine regulation, consistent with the clinical feature of hormonal metabolic disorder commonly observed in PNALD patients (49). Additionally, abnormal glyoxylate metabolism may be associated with hepatic oxidative stress as glyoxylate accumulation exacerbates oxidative damage to hepatocytes by inducing the production of reactive oxygen species (50). Three core candidate metabolites were identified through multi-algorithm screening. Among them, α-teresantalic acid was upregulated in the PNALD group, while 6-n-octylaminouracil and 6β-hydroxy-hydromorphone were downregulated. Treatment with 6-n-octylaminouracil significantly inhibits tumor growth and enhances the anti-tumor effect of anti-PD-1 therapy (51). Currently, there are no studies directly investigating the relationship between α-teresantalic acid or 6β-hydroxy-hydromorphone and PNALD. The detection of 6β-hydroxyhydroxymorphine without exogenous opioid administration suggests that it may not result from direct contamination but rather from structural analogs produced by disrupted endogenous steroid or gut microbial metabolic networks during parenteral nutritional stress conditions. Such “drug-like” molecules generated by host or microbial metabolic disorders have been reported in other metabolic diseases (52). However, the causal relationship between these two metabolites and PNALD remains unclear. Whether they act as drivers of liver injury, incidental byproducts, or protective responsive molecules should be verified in gain- and loss-of-function experiments. Elucidating their specific roles in the pathophysiology of PNALD will be an important direction for developing new treatments.

To analyze the holistic pathological mechanism of PNALD, this study further constructed association networks involving genes, metabolites, and immune cells. The five core biomarkers (*Itgam, Clec4d, Orm2, Lcn2*, and *Cd14*) all showed significant correlations with three candidate metabolites, by which inflammatory markers regulate metabolite synthesis. For example, *Lcn2* could affect the TCA cycle by regulating iron metabolism, thereby altering α-teresantalic acid (53). Immune infiltration mediated by *Itgam* may inhibit the activity of hepatic drug-metabolizing enzymes through inflammatory signals, leading to reduced accumulation of 6β-hydroxy-hydromorphone (54). Furthermore, GSEA analysis revealed that the core biomarkers were enriched in cytokine-cytokine receptor interaction and oxidative phosphorylation pathways. Cytokines exert biological effects by binding to specific receptors, including inducing cell apoptosis, regulating cell development and differentiation as well as immune responses, mediating inflammatory reactions, and promoting tissue repair (55, 56). A study found that differential proteins in PNALD were mainly associated with mitochondrial oxidative phosphorylation, and proposed that mitochondrial energy metabolism disorders, hepatic glucose and lipid metabolism disturbances, and excessive oxidative stress damage might constitute the comprehensive mechanism of PNALD (2). In the association analysis between biomarkers and immune cells, this study identified 19 DICs. Among them, monocytes showed high infiltration in both groups of samples, and MDSCs exhibited strong positive correlations with all five core biomarkers. MDSCs are a type of cell that exhibits immunosuppressive activity. They not only alleviate inflammation by secreting IL-10 and TGF-β during liver injury, but also participated in the pathological mechanisms of various chronic liver diseases, such as chronic viral hepatitis, alcoholic liver disease, NAFLD, and autoimmune liver disease (57–59). This strong association suggested a potential balance mechanism involving inflammatory activation and immune compensation in PNALD. For example, while *Lcn2* activates inflammation, it might also recruit MDSCs to restrict excessive inflammatory development and prevent further hepatocyte damage (60). This finding indicated that MDSCs may influence the progression of liver diseases through immunomodulatory effects.

To promote the translation of core biomarkers into clinical applications, their upstream regulatory molecules and potential therapeutic drugs were further predicted. Transcription factor Nfkb1, a core component of the NF-κB pathway, regulated both *Itgam* and *Lcn2*, playing a key regulatory role in hepatic inflammation (61, 62). Mmu-miR-466j may target *Itgam* and *Clec4d*, and abnormal expression of miRNA resulted in the upregulation of target genes, thereby exacerbating inflammation (63). Furthermore, the association between lncRNA Taf4 and related miRNAs suggested that the lncRNA-miRNA-mRNA regulatory network was involved in the molecular regulation of PNALD. In terms of potential therapeutic drugs, this study identified drugs such as liarozole (targeting *Itgam*), IC14 (targeting *Cd14*), and anhydrous docetaxel (targeting *Orm2*). Liarozole is a cytochrome P450 inhibitor, and existing studies have shown that it can alleviate cholestasis by regulating liver metabolism (64). IC14 is a TLR4 pathway inhibitor that can inhibit *Cd14*-mediated inflammatory activation (65). These predicted drugs provided a candidate library for the targeted therapy of PNALD, and their efficacy and safety will be further verified through *in vitro* and *in vivo* experiments.

To date, the academic community has developed a variety of models to simulate distinct pathological dimensions of PNALD. The *in vitro* buffalo rat liver cell model treated with soybean oil emulsion can be used to study the role of endoplasmic reticulum stress in lipid accumulation (66). The *in vivo* models are primarily established in rodents. Rat models are mostly used for dissecting cholestasis and aluminum contamination-induced hepatotoxicity, as well as for testing interventional strategies (67, 68). Mouse models are more suitable for genetic studies (69). Large animal models, such as piglets and newborn rabbits, have significant value for the study of related pathological mechanisms because their physiological characteristics are similar to those of humans (70, 71). Although various models have unique advantages for mechanistic dissection, human relevance, genetic manipulability, and physiological similarity, the multi-omics framework of the present study provides a unique opportunity for cross-scale integration of molecular mechanisms. It should be noted that there are differences between animal models and human pathologies. Therefore, further validation in clinical cohorts and more complex pathological models is warranted to facilitate the clinical application of our findings.

Although this study systematically analyzed the pathological mechanism of PNALD, there are still certain limitations. Specifically, we have highlighted the correlation between key metabolites and the dysregulation of lipid metabolism and inflammatory signaling, as well as their potential implications for the gut microbiota-liver axis. However, we acknowledge that the current interpretation of the phenotype-molecule-immunity regulatory network remains at the level of correlation, and further mechanistic evidence is needed to establish causal relationships. The study was conducted based on a mouse PNALD model, whose etiology differs from that of humans. The expression of candidate biomarkers was currently verified using a single method. We fully recognize the limitations of the current study, including the reliance on a mouse model, the lack of functional validation at the cellular/animal level, and the insufficient connection to clinical practice. We are committed to further in-depth exploration in follow-up studies, including the collection of human PNALD samples for biomarker validation, the establishment of gene intervention models to clarify causal mechanisms, and the integration of gut microbiota analysis to bridge the gap with clinical translation.

## 5 Conclusion

This study systematically analyzed the hepatic pathological features and molecular regulatory mechanisms of PNALD using a mouse model. We pathological morphology, functional detection, multi-omics analysis, and correlation verification. PNALD was significantly induced by hepatic pathological damage and caused functional abnormalities in mice. RNA-seq identified *Itgam, Clec4d, Orm2, Lcn2*, and *Cd14* as core biomarkers of PNALD. Meanwhile, metabolome analysis identified three core candidate metabolites, including 6-n-octylaminouracil, 6β-hydroxy-hydromorphone, and α-teresantalic acid. This study not only clarified the core pathological characteristics and molecular biomarkers of PNALD, but also constructed a phenotype-molecule-immunity regulatory network, laying a solid foundation for mechanistic studies of PNALD as well as the development of clinical diagnosis and intervention strategies.

## Data Availability

The original contributions presented in the study are included in the article/Supplementary material, further inquiries can be directed to the corresponding author.
