# Supplementary material for: Integrated transcriptomics–metabolomics analysis reveals biomarkers and metabolic dysregulation characteristics of parenteral nutrition–associated liver disease
Source: Front Nutr. 2026 Mar 31;13:1760274. doi: 10.3389/fnut.2026.1760274 (PMC13076265; doi:10.3389/fnut.2026.1760274)
Supplement: Supplementary file 1 [file Data_Sheet_1.docx]

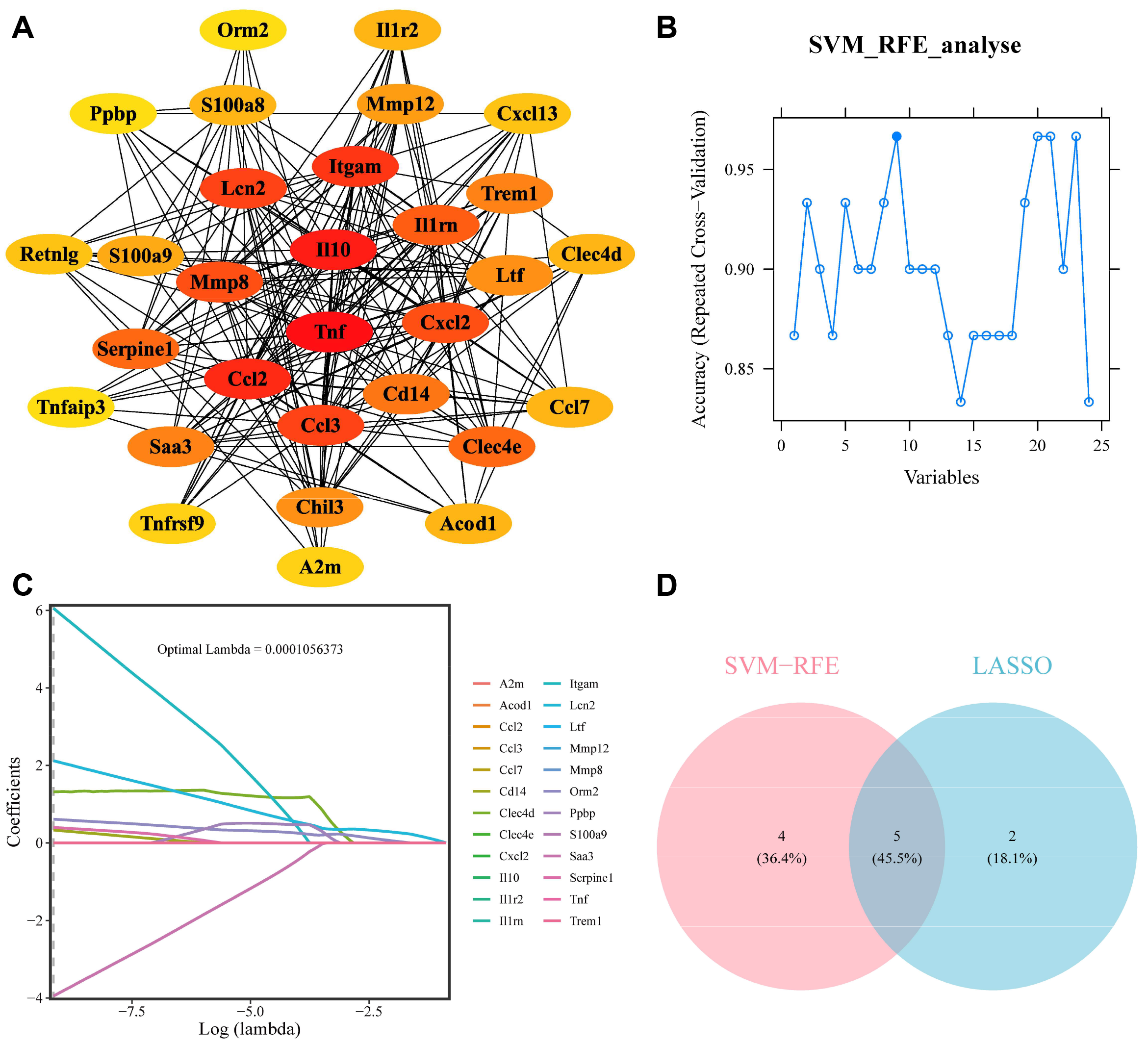


**Supplementary Figure 1. Biomarker identification in PNALD. A.** Protein-protein interaction network showing interactions among differentially expressed genes (DEGs), with nodes representing genes and edges representing interactions. **B.** Accuracy of repeated cross-validation in the support vector machine-recursive feature elimination (SVM-RFE) analysis, with the x-axis indicating the number of variables and the y-axis representing accuracy. **C.** Coefficients of genes in the least absolute shrinkage and selection operator (LASSO) analysis, with the x-axis being log(lambda) and the y-axis showing coefficients. D. Venn diagram depicting the intersection of gene sets identified by SVM-RFE and LASSO algorithms. PNALD, pediatric nonalcoholic fatty liver disease.


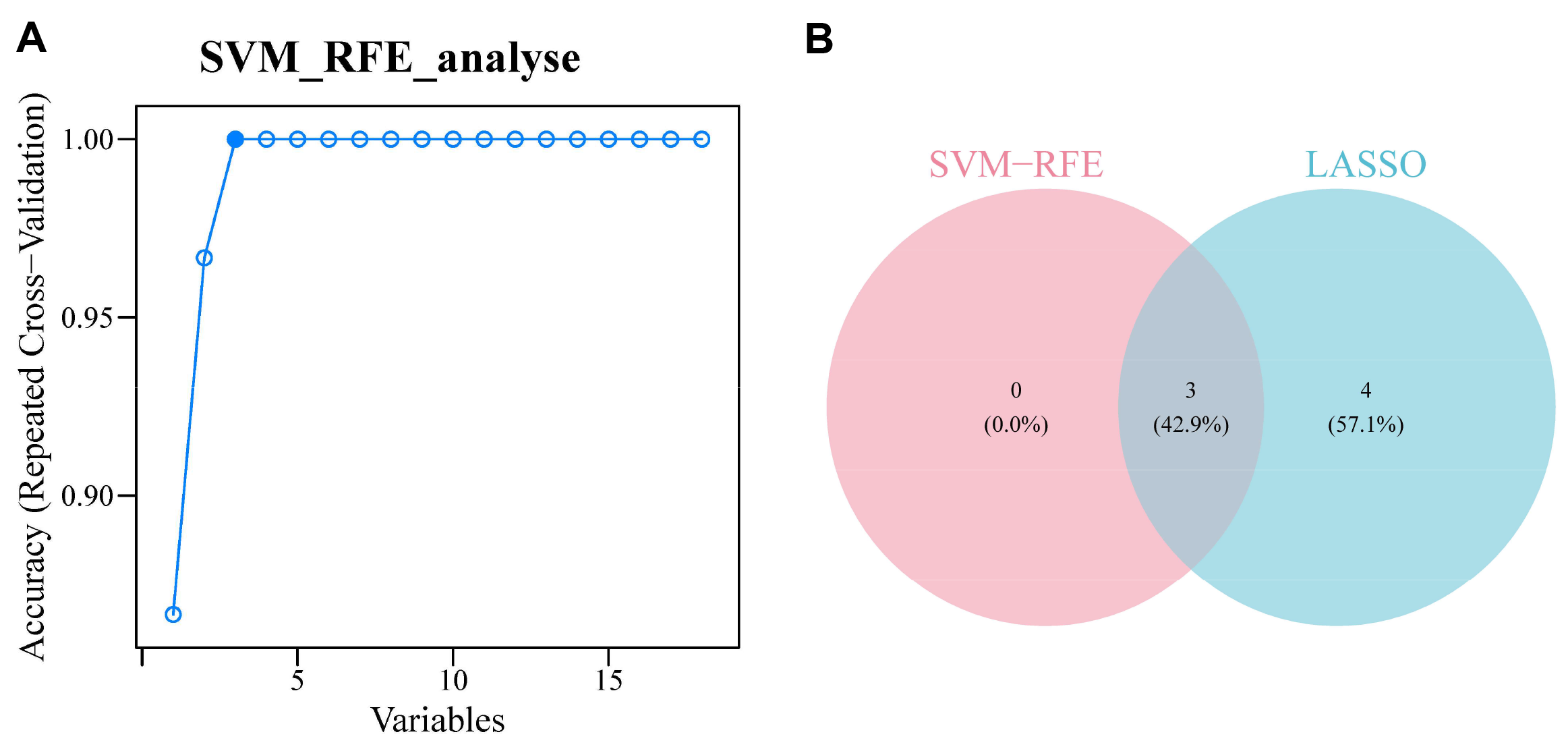


**Supplementary Figure 2. Metabolite identification in PNALD.** **A.** Accuracy of repeated cross-validation in the support vector machine-recursive feature elimination (SVM-RFE) analysis, with the x-axis indicating the number of variables and the y-axis representing accuracy. **B.** Venn diagram depicting the intersection of metabolite sets identified by SVM-RFE and LASSO algorithms. PNALD, pediatric nonalcoholic fatty liver disease.


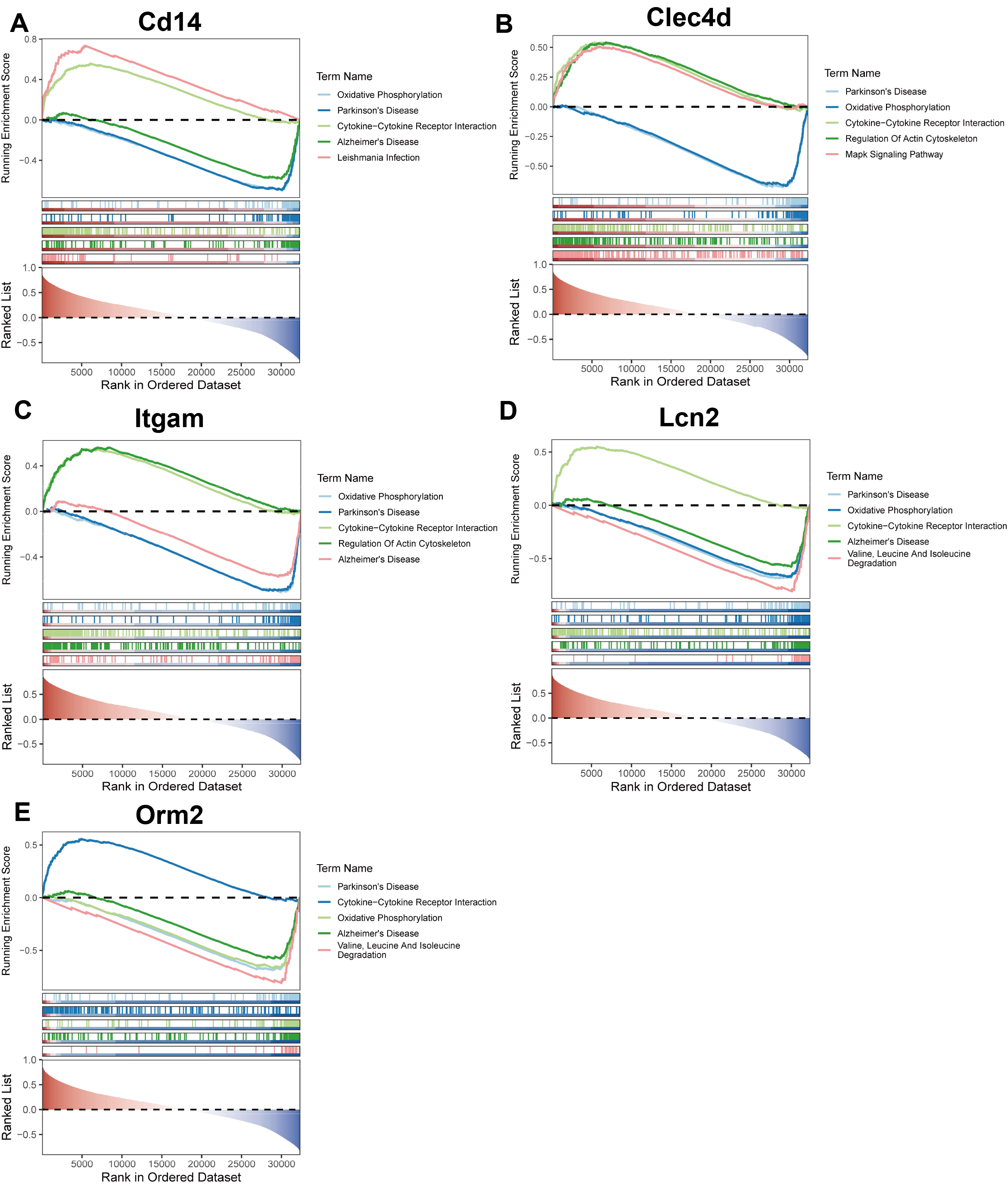


**Supplementary Figure 3. Gene set enrichment analysis (GSEA) of key biomarkers in PNALD. A-E.** GSEA plots for biomarkers *Cd14* (A), *Clec4d* (B), *Itgam* (C), *Lcn2* (D), and *Orm2* (E), respectively.


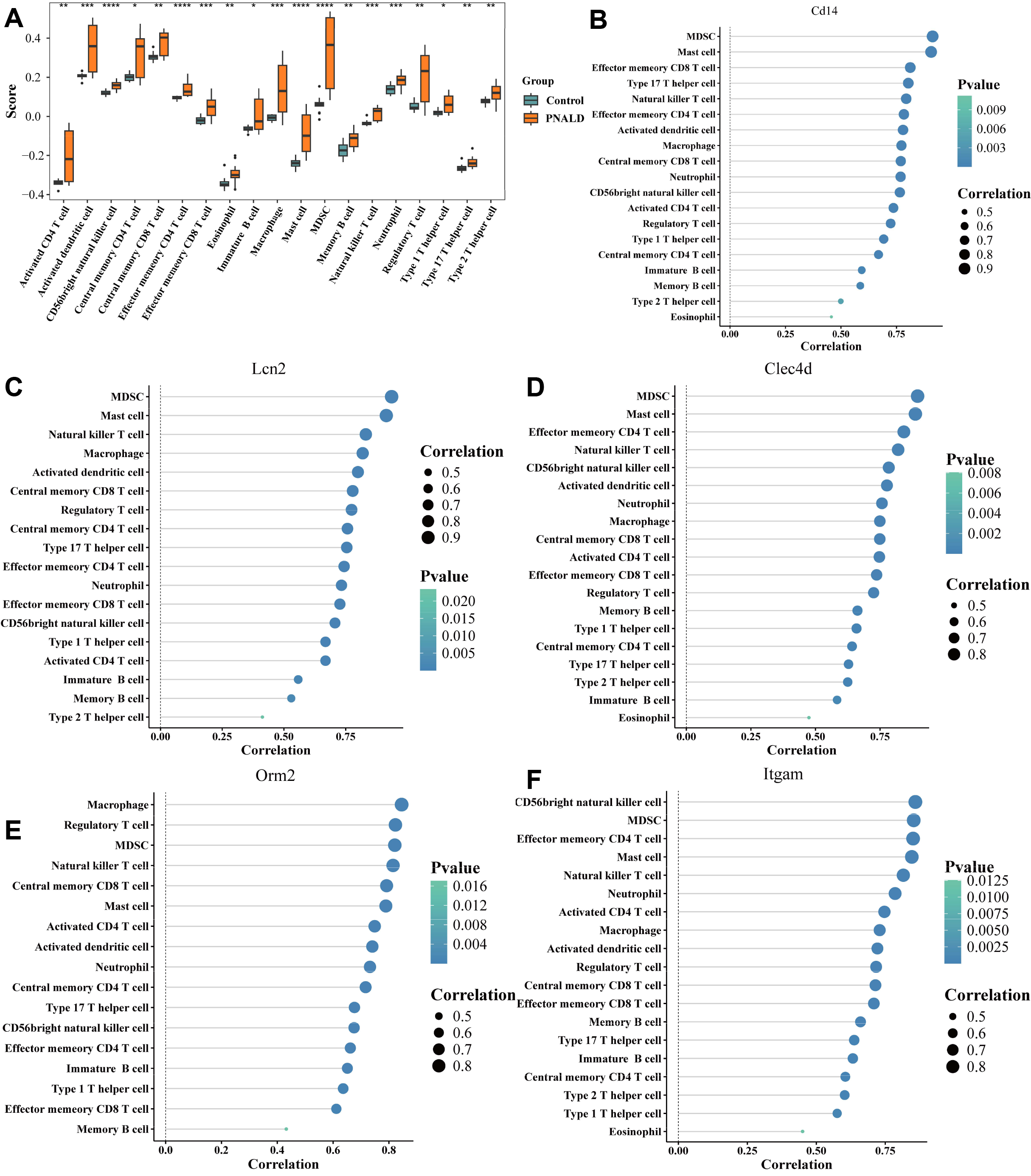


**Supplementary Figure 4: Correlation between biomarkers and differentially infiltrating immune cells. A.** Identification of differentially infiltrating immune cells (DICs) between PNALD and control samples, with a boxplot showing the score distribution of these DICs. **B-F.** Correlation analysis between biomarkers and DICs, with the x-axis representing correlation strength, the y-axis listing immune cell types, and color indicating P value.


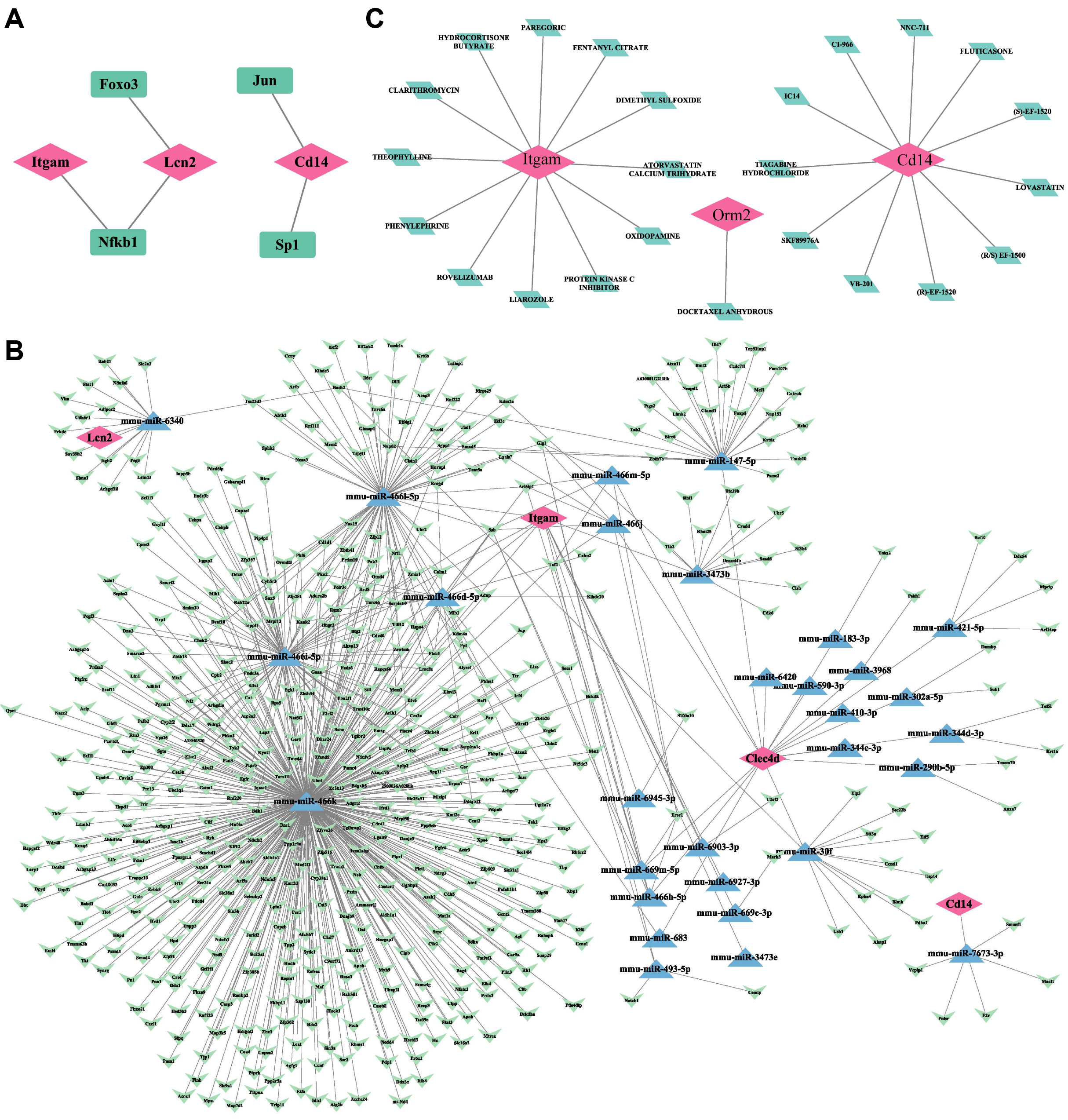


**Supplementary Figure 5. Regulatory networks and drug prediction related to key biomarkers in PNALD. A.** Transcription factor (TF)-biomarker regulatory network. Nodes represent TFs (green) and biomarkers (pink), and edges represent regulatory links. **B.** Competing endogenous RNA network involving long non-coding RNAs (lncRNAs), microRNAs (miRNAs), and biomarkers. Nodes represent lncRNAs (green), miRNAs (blue), and biomarkers (pink), and edges represent interactions. **C.** Drug-biomarker association network. Nodes represent drugs (blue) and biomarkers (pink), and edges represent predicted associations.
